# Balancing the presentation of information and options in patient decision aids: an updated review

**DOI:** 10.1186/1472-6947-13-S2-S6

**Published:** 2013-11-29

**Authors:** Purva Abhyankar, Robert J Volk, Jennifer Blumenthal-Barby, Paulina Bravo, Angela Buchholz, Elissa Ozanne, Dale Colins Vidal, Nananda Col, Peep Stalmeier

**Affiliations:** 1 Nursing, Midwifery and Allied Health Professions Research Unit, University of Stirling, Unit 13 Scion House, Stirling University Innovation Park, Stirling, FK9 4NF; 2 Department of General Internal Medicine, Unit 1465, The University of Texas MD Anderson Cancer Center, 1515 Holcombe Blvd, Houston, Texas 77230, USA; 3 Center for Medical Ethics and Health Policy, Baylor College of Medicine, One Baylor Plaza, MS: BCM420, Houston, Texas 77030-3411, USA; 4 Department of Woman’s Health, School of Nursing, Pontificia Universidad Catolica de Chile, Post code 7820436, Chile; 5 Institute of Medical Genetics, School of Medicine, Cardiff University, Post code CF14 4YS, UK; 6 Department of Medical Psychology, University Medical Center Hamburg-Eppendorf, Martinistraße 52, D - 20246 Hamburg, Germany; 7 Institute for Health Policy Studies, University of California, 3333 California Street, San Francisco, CA 94118, USA; 8 Department of Surgery, Dartmouth-Hitchcock Medical Center, One Medical Center Drive, Lebanon, NH 03766, USA; 9 The Dartmouth Institute for Health Policy and Clinical Practice, Geisel School of Medicine at Dartmouth, 35 Centerra Pkwy, Lebanon, NH 03766, USA; 10 College of Osteopathic Medicine, University of New England, Biddeford Campus, 11 Hills Beach Road, Biddeford, Maine 04005, USA; 11 Center for Excellence in the Neurosciences, University of New England, Biddeford Campus, 11 Hills Beach Road, Biddeford, Maine 04005, USA; 12 Department of Epidemiology, Biostatistics and HTA, Radboud University Nijmegen Medical Centre, Nijmegen, The Netherlands

## Abstract

**Background:**

Standards for patient decision aids require that information and options be presented in a balanced manner; this requirement is based on the argument that balanced presentation is essential to foster informed decision making. If information is presented in an incomplete/non-neutral manner, it can stimulate cognitive biases that can unduly affect individuals’ knowledge, perceptions of risks and benefits, and, ultimately, preferences. However, there is little clarity about what constitutes balance, and how it can be determined and enhanced. We conducted a literature review to examine the theoretical and empirical evidence related to balancing the presentation of information and options.

**Methods:**

A literature search related to patient decision aids and balance was conducted on Medline, using MeSH terms and PubMed; this search supplemented the 2011 Cochrane Collaboration’s review of patient decision aids trials. Only English language articles relevant to patient decision making and addressing the balance of information and options were included. All members of the team independently screened clusters of articles; uncertainties were resolved by seeking review by another member. The team then worked in sub-groups to extract and synthesise data on theory, definitions, and evidence reported in these studies.

**Results:**

A total of 40 articles met the inclusion criteria. Of these, six explained the rationale for balancing the presentation of information and options. Twelve defined “balance”; the definition of “balance” that emerged is as follows: “The complete and unbiased presentation of the relevant options and the information about those options—in content and in format—in a way that enables individuals to process this information without bias”. Ten of the 40 articles reported assessing the balance of the relevant decision aid. All 10 did so exclusively from the users’ or patients’ perspective, using a five-point Likert-type scale. Presenting information in a side-by-side display form was associated with more respondents (ranging from 70% to 96%) judging the information as “balanced”.

**Conclusion:**

There is a need for comparative studies investigating different ways to improve and measure balance in the presentation of information and options in patient decision aids.

## Background

Current guidelines outlining the quality standards for patient decision aids (PtDAs) require that PtDAs present the information and options in a balanced manner [1]. However, these guidelines provide little explanation and elaboration about what constitutes a “balanced presentation”, why balance is essential for informed patient decision making, what strategies developers use to present information in a balanced manner, nor how to assess the balance of information and options during the development or evaluation of PtDAs. The purpose of this review was to update the theoretical justification and empirical evidence for balancing the presentation of information and options within PtDAs, as proposed by the International Patient Decision Aid Standards (IPDAS) Collaboration.

The specific objectives of the review were to:

• Identify the attributes of information presentation that constitute “balance” and explain the theoretical basis for balancing the presentation of information and options in PtDAs.

• Determine whether the balance of information and options is assessed in empirical studies of PtDAs and, if so, how it is assessed.

• Assess the impact of presenting the information in a side-by-side display format on ratings of balance.

• Identify and discuss the emerging issues related to the definition, theoretical rationale, and empirical evidence about the balanced presentation of information and options in PtDAs.

## Review methods

An international, multidisciplinary team of nine researchers working in the area of PtDAs was convened to update the theoretical and empirical basis for balancing the presentation of information and options through a review of literature.

### Search strategy

The search strategy was developed with reference to the review aims and was guided by the expert advice of a librarian with training in literature search, retrieval, and review. The electronic databases Ovid MEDLINE and PubMed (1980 to October 2011) were searched for articles that reported information on the balance of PtDAs, using the following Medical Subject Headings (MeSH) terms: (decision aid* or decision support) *and* (equitabl* or balanc* or neutral* or bias* or slant* or inequitabl* or unbias* or unbalanc*). In addition, all 86 articles included in the 2011 Cochrane Collaboration’s systematic review of randomized controlled trials of PtDAs, plus an additional 10 studies that were included in the Cochrane Registry of Decision Aids [2], were reviewed for any mention of the above search terms.

### Inclusion and exclusion criteria

Articles were included if they were relevant to patient decision making, were published in English, and addressed the balance/bias of information and options. Exclusion criteria were: publication prior to 1980, not peer reviewed, conference proceedings, and articles relevant to physician-targeted decision support (as opposed to patient decision support). No exclusions were made based on study type; thus, systematic reviews and primary studies without a control group (e.g., feasibility studies) were also included.

### Selection of articles

The titles and abstracts identified by the search strategies were evenly distributed among seven members of the review team. Articles in each cluster were evaluated on the basis of title and abstract by a single member of the team applying the inclusion and exclusion criteria. Articles were coded as “Accept for Review” (clear evidence that balance/bias of patient decision aids or other patient educational materials is addressed); “Reject” (no evidence that balance/bias of patient decision aids or other educational materials is addressed); or “Unsure” (agreement to be established with another member of the group/decision to be made after full text review).

**Figure 1:**
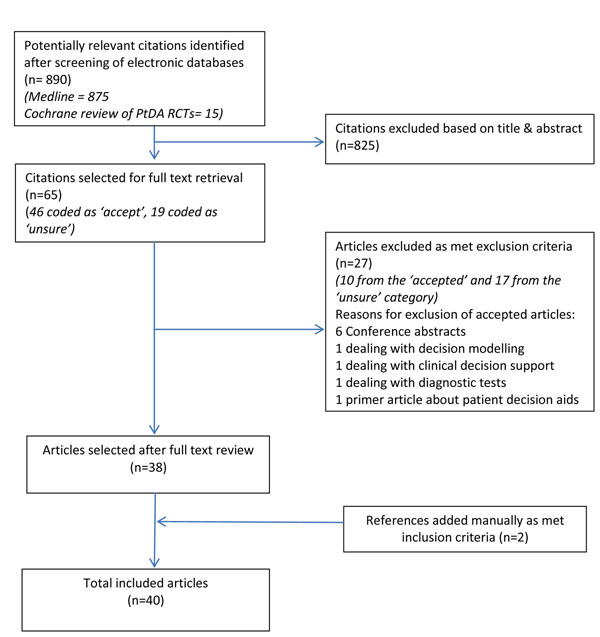
Study selection process

Full manuscripts were retrieved for articles coded as “accept” or “unsure”. Each team member discussed articles coded as “unsure” with another team member. If uncertainty remained, a third member (PS) reviewed the full paper to reach a decision about its inclusion/exclusion. All the accepted articles were screened by PS to ensure that they fulfilled the inclusion criteria. The numbers of papers identified, screened, and excluded at each step in this study selection process is illustrated in Figure 1. Authors were contacted if their PtDA could not be found; two authors did not respond.

### Data extraction

For each paper, the names of the author(s), the year of publication, and the decision context were noted. Then the research team was divided into two review groups: a definitions and theory group, and an empirical evidence group. Each group developed and systematically applied a data extraction form to the selected articles

The *definitions and theory group* collected two data sets. The first captured whether or not the article under review used any terms for balance, and, if so, whether any definition for balance was provided. The second data set focused on whether or not the article provided a theoretical rationale for balancing the presentation of information and options.

The *empirical evidence group* undertook several data-collection steps. In the first step, they identified whether or not a published paper reported that the balance of the relevant PtDA was assessed. Then, in their second step, among those articles in which balance was assessed, they collected data about: a) the sample and sample size used to assess balance; b) how the assessment of balance was carried out; c) the response options from the measure of balance; d) whether or not the PtDA presented the information in a side-by-side display format (e.g., by using side-by-side graphics, side-by-side tables, or side-by-side bulleted information) designed to enable users to compare and contrast the options; and e) the reported percentage of respondents who rated the PtDA as balanced.

### Data summary and synthesis

In the case of the definitions and theory group, data summary and synthesis yielded an updated definition for “balance”, and an overarching theoretical rationale for evaluating PtDAs according to this quality dimension (see below).

In the case of the empirical evidence group, data summary and synthesis proceeded along two paths. The first path involved considering the overall evidence about balancing the presentation of information and options in PtDAs. Accordingly, the group computed descriptive statistics about the numbers of articles that did/did not report whether the balance of the relevant PtDA was assessed, as well as about the sample sizes, the measures used, and the basic results of those assessments when they were carried out.

On the second analytic path, the empirical evidence group focused more closely on the articles that reported on the assessment of balance. A common approach to presenting information in PtDAs is to summarize the attributes of each option in a side-by-side display format. Therefore, we were interested in exploring the potential relationships between side-by-side presentations of information and subsequently-reported assessments of balance. Accordingly, the group sub-categorized these as a) studies in which respondents received a PtDA *with* a side-by-side presentation of information designed for comparing and contrasting the options, and b) studies in which respondents received a PtDA *without* side-by-side presentation. We performed additional analyses on these sub-categories—using a random effects model to take into account heterogeneity across studies—to compare them in terms of the percentages of patients who agreed that the relevant decision aid was balanced. We used univariate analysis of variance (ANOVA), which used weighted least squares, and again weighted by the number of patients in the samples. With this conservative method of weighting, the analysis was based on the number of studies in a “category”, and not on the number of patients included. All analyses were performed with SPSS (version 18.0).

## Review results

Forty articles were included in this review; 38 were identified from the database searches and two additional articles (co-authored by a member of the research team) were added manually after being judged as meeting the inclusion criteria [1,3-41]. The findings relating to the definition, theoretical rationale, and empirical evidence are presented below.

## “Balancing the presentation of information and options”: a definition

The relevant chapter in the original 2005 IPDAS Background Document [42] was entitled “balancing the presentation of options”. As we conducted the review reported here, we judged this original title as too narrow in scope, because it is limited to balancing the type and number of “options” presented (i.e., which and how many options), and does not extend to the balancing of information presented within each option. Given that the literature highlights the importance not only of complete, balanced, and unbiased presentation of all available options but also complete, balanced, and unbiased presentation of information about all aspects of those options [5,6,10], we broadened the title for this quality dimension to “balancing the presentation of information and options”.

Furthermore, no explicit definition of “balance” was provided in the original chapter [42]. There was an implication that information presented in a balanced manner is that which does not inadvertently persuade the user to accept or reject a particular option. However, this implication only points to the purpose of balanced presentation within PtDAs; it is unlikely to be useful for developers of PtDAs because it does not specify the attributes of information presentation that constitute a “balanced manner”.

Accordingly, in our review we sought to identify the attributes of balanced presentation. Of the 40 articles, 12 defined balance, explicitly or implicitly. (The remaining articles did not define balance, although some included superficial findings regarding the perceived balance of information among users.) The attributes of balanced presentation identified by this literature review process were integrated to provide the following definition: *“Balance” refers to complete and unbiased presentation of the relevant options and the information about those options - in content and in format - in a way that enables individuals to process this information without bias.*

This definition has four characteristics. First, it includes the attribute “complete”, which refers to the presentation of all the relevant options (which, in turn, may include the option of “doing nothing”) and to the presentation of information on all aspects of those options (i.e. risks, benefits, uncertainties, procedures, consequences). Secondly, it includes the attribute “unbiased”—also referred to as “neutral” or “non-directive”—which refers to presentation in a way that places equal weight on positive and negative information and avoids placing a value judgement on that information. Thirdly, this definition highlights that “balance” applies to the content of information (i.e. what information is presented) as well as the format of information (i.e. how it is presented). Format of information is an umbrella term that includes both the display format (e.g. side-by-side versus linear, graphs/pictographs versus text) as well as the probability format (absolute versus relative risks, positive versus negative frames). Finally, it refers to the impact of presentation on individuals’ information processing (i.e. their gaining of knowledge, their formulation of risk and benefit perceptions, and their construction of preferences).

## Theoretical rationale for evaluating patient decision aids on this quality dimension

The theoretical rationale for evaluating PtDAs in terms of how well they balance the presentation of information and the presentation of options was developed by integrating the review findings with psychological theory. Of the 40 articles included in this review, a rationale for balancing the presentation of information and options was provided in six [8-13], none of which referred to any specific theory or framework (See Additional file 1 Table S1). However, three of these six articles [9-11] explained the rationale in terms of the cognitive processes that are affected by the presentational aspects of the information rather than its actual content. The review findings were synthesised in light of the broader information-processing paradigm that encompasses the cognitive processes referred to in the theoretical rationale provided in each article.

The basic purpose of a PtDA is to foster informed decision making, by improving the understanding of risk and benefits of options, improving the comprehension of probability information, creating more realistic expectations about the consequences of options, and improving clarity about personal values. An informed decision is made when individuals a) take into account the consequences of all the available options, b) assess the likelihood and value of those consequences without bias, and c) make trade-offs between these evaluations [43,44].

This requires significant cognitive resources and effort. As human beings, we have finite cognitive resources for acquiring, storing, retrieving, and processing decision-related information [45]. As a result, we employ two types of strategy to process information [46].

Heuristic processing (“system 1”) strategies involve unconscious “rules of thumb” that often are triggered by different cues in the environment/context of the decision-related information. These strategies include, for example, evaluating only a part of the information, screening out information based on initial impressions, settling on the first option that appears to be satisfactory, or using cues from the informational context to make a choice. This requires little cognitive effort, and is less time-consuming.

Systematic processing (“system 2”) strategies involve deliberative analytical approaches. These include, for example, evaluating all possible consequences of all options, accurately appraising their likelihood and desirability, and making trade-offs. This requires considerably more conscious effort and is relatively time-consuming.

For decisions that involve some degree of uncertainty or difficult trade-offs, individuals have a natural preference for using the heuristic strategies, as they minimise the load on their cognitive resources. This means that they are more likely to be influenced by the subtle cues in the context of the information (e.g., how information is presented) rather than by the content of the information (such as the risks and benefits of options) [45,47]. Therefore, an unbalanced presentation of uncertain or complex information, in conjunction with the favoured use of heuristic strategies, can compromise individuals’ ability to engage in systematic processing. For instance, if information is incomplete, people will, by default, ignore missing information, which may lead them to inadvertently overvalue or devalue a treatment option [10]. If information is presented in a non-neutral manner, it can stimulate a range of cognitive biases that can unduly affect people’s knowledge, their perceptions of risks and benefits, and, ultimately, their preferences [9]. These untoward effects would, in turn, undermine the achievement of the PtDA’s purpose. For these reasons, it is important that PtDAs present all the relevant options and the information about those options in a complete, unbiased and neutral manner that is sustained throughout the PtDA’s content and format

## Empirical evidence on the balance of information and options within PtDAs

Among the 40 included papers, 11 studies reported an assessment of balance, but only 10 studies involved at least 10 respondents (see Table 1, column 1). Findings on the ways of assessing balance and the impact of using side-by-side presentation on balance ratings are summarised below.

**Table 1:** Ten studies reporting respondents’ ratings of the balance of a patient decision aid

Author, Year, Citation	Sample size*	Respondents who reported the PtDA was balanced. n (%)	Did the PtDA include side-by-side display of evidence?	Did the PtDA include any side-by-side display?
Mathieu, Barratt et al., 2010 [14]	117	66 (57%)	no	no
Smith, Trevena et al., 2010 [15]	334	160 (48%)	no	no
Griffith, Fichter et al., 2008 [6]	106	17 (16%)	unclear	unclear
Spunt, Deyo et al., 1996 [16]	239	133 (56%)	unclear	unclear
Anderson, Carter et al., 2011 [17]	19	17 (89%)	no	yes
Watson, Hewitson et al., 2006 [18]	468	439 (94%)	no	yes
Drake, Engler-Todd et al., 1999 [19]	38	27 (71%)	yes	yes
Lalonde, O’Connor et al., 2004 [20]	16	13 (80%)	yes	yes
van Tol-Geerdink, Stalmeier et al., 2006 [21]	150	142 (95%)	yes	yes
van Tol-Geerdink, Leer et al., submitted [22]	153	147 (96%)	yes	yes

*Only studies with at least 10 subjects were included.

### Whether and how assessment of balance was carried out

In the 10 papers that involved at least 10 respondents, balance of the PtDA was exclusively assessed from the users’/patients’ perspective. Studies tended to ask respondents to assess balance by using a five-point Likert-type scale either a) to rate the strength of agreement/disagreement in response to a question about the presentation being balanced, or b) to rate the extent to which the information was slanted/favoured a particular option (or not) (see Additional file 2 Table S2).

For each of the 10 papers, we computed the percentage of respondents who found the relevant PtDA “completely balanced” (Table 1, columns 2 and 3). This percentage ranged from 16% to 96%. Six PtDAs were rated as completely balanced by at least 70% of the respondents.

### The impact of using side-by-side display format to contrast the options

Six out of ten studies reported presenting the information about options in a side-by-side display format, two included no such format, and the presence of such a format remained unclear for the remaining two. Of the six studies that included a side-by-side display format, four included “side-by-side display of evidence” and two included “side-by-side display of arguments”.

*Side-by-side display of evidence* involves presenting numeric probabilistic information about the possible consequences of options in the form of a column/row matrix. Such display formats contain information about options along the columns and information about possible consequences along the rows. The cells contain the probability of each outcome (e.g., “x out of 100 experience (…the outcome…)”, either with or without a visual aid to depict x out of 100. See van Tol-Geerdink, 2006 [21] for an example.

*Side-by-side display of arguments* involves presenting, in a matrix form, verbatim descriptions of options, treatment processes, their possible consequences, and/or reasons for choosing and not choosing the options. Similar to s*ide-by-side display of evidence*, this display contains information about options along the columns and information about their attributes along the rows. The cells contain usually verbatim information on, for example, option descriptions, processes, consequences, and reasons. Numeric information, if present, does not systematically contrast the options according to the probabilities of their outcomes.

**Figure 2:**
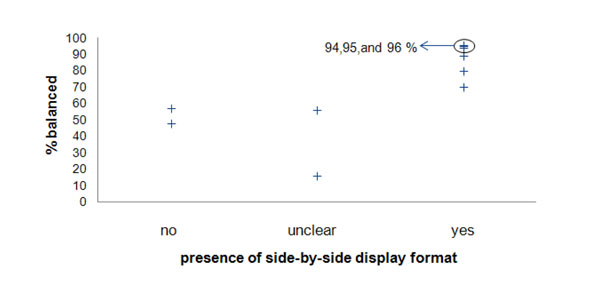
Percent of respondents rating a patient decision aid as balanced, stratified by use of a side-by-side display contrasting the options

The results of the analyses comparing the six studies of PtDAs that included a side-by-side display and the four studies of PtDAs that did not include a side-by-side display are depicted in Figure 2.

The results show that PtDAs including any side-by-side display (either of evidence or arguments) received the highest ratings as being “balanced”, compared to PtDAs that included no side-by-side display of information (F(2,7) = 21.18, *p* = 0.001). Thus, the presentation of information in a side-by-side display format in which the options can be compared was associated with more respondents (ranging from 70% to 96%) judging the information as “balanced”. The results also show that the side-by-side display of *evidence*, in which the options can be compared using probabilistic outcome information, was not associated with respondents being more or less likely to rate an aid as “balanced”, compared to the side-by-side display of *arguments* (F(2,7) = 2.77, *p* = 0.130). Side-by-side display of evidence was consistently rated as “balanced” by more than 70% of the respondents (see Table 1). These findings are consistent with evidence from other research suggesting that side-by-side presentation of information leads to greater understanding of probabilistic information as compared to presentation of information in a linear format [48,49].

In summary, our review of the empirical evidence suggests that PtDAs that include side-by-side presentation of information aimed at facilitating the comparison of attributes within and across the different options, are more likely to be perceived as “balanced” than those which do not include such display formats. However, there is insufficient evidence for the superiority of “side-by-side display of evidence” at achieving balance over “side-by-side display of arguments”. The findings, therefore, must be interpreted with caution. These results simply imply that the presentation of information in a side-by-side display format (compared to a linear display format) is more likely to be perceived as “balanced”; they do not objectively assess the balance in the content or in the probability format of the information.

## Discussion: some emerging issues/research areas

There are several debatable issues about balancing the presentation of information and options; further research is required in these areas.

### Deviating from neutrality in order to counter pre-existing biases

There is debate about whether PtDAs should always strive for neutrality or, in some situations, attempt to counter or undo known biases [50]. We acknowledge that the provision of PtDAs does not occur in a vacuum. Patients and clinicians bring to the table a wide range of pre-existing values and beliefs, which may stem from misinformation, previous experience, or heuristic thinking or practice norms [51,52]. It has been argued that, in certain situations, it is ethically appropriate for PtDAs not to be balanced—insofar as they are countering an existing bias—in order to bring the patient to an overall balanced perspective [50]. In such circumstances, PtDAs could nudge patients towards particular options by, for example, bringing those options to the forefront, describing misconceptions or non-evidence-based practice patterns, or presenting patients with data about what others with similar goals, habits, or concerns choose (i.e., providing descriptive norms).

Others argue that the goal of PtDAs is to enable patients to make informed decisions by helping them to carry out a balanced consideration of the options in a manner that minimises the biases resulting from pre-existing beliefs, perceived norms, contextual cues, or different styles in which information is presented [53]. The design of PtDAs involves several elements intended to limit or minimise the influences that these biases have on patients’ judgements and choices [53,54]. The balanced presentation of options and information is one such element of PtDA design that may limit or even counter the influence of biases by encouraging people to attend to all the pertinent information. However, it remains to be empirically established whether balanced presentation alone is sufficient to counter preexisting biases. Misconceptions or non-evidence-based practice norms may be addressed in a separate section of a PtDA’s design, independent of the main information content, without compromising the balance of information.

For health promotion situations in which clinical evidence supports particular treatment/screening options as being high-benefit and low-risk, the appropriate motivation may be to use an intervention that is aimed at promoting informed uptake, rather than informed decision making [54,55]. The content and structure of information interventions should match their purpose [54]; interventions aimed at enabling informed decision making should always strive for balance, whereas those aimed at promoting informed uptake may include nudging techniques. Perhaps, for the latter purpose, the term “health promotion tool” instead of “patient decision aid” should be used, to avoid unnecessary confusion about the primary goal of decision aids—that is, the impartial provision of information.

### Provision for the inclusion of a “do nothing” option

The “balance” definition proposed in this paper includes a provision that the presentation of the “do nothing” option may be necessary for a balanced PtDA. Whether PtDAs should always include a default “do nothing” option is currently under debate. We recognize that, for some health-related decisions, it would seem odd to offer “doing nothing” as an option, such as in cases in which “doing nothing” is not supported by evidence, in which the expert community’s opinion is to treat in one way or another, or in which the patient wants a particular treatment. One reasonable response to this situation is to acknowledge that PtDAs should provide information about the consequences of doing nothing while not presenting it as a reasonable option. Giving information about the consequences of doing nothing is what is ethically important, as outlined by The American Medical Association statement on Informed Consent [56].

### Challenges for developing balanced decision aids

The “balance” definition proposed in this paper poses particular challenges to those developing PtDAs, in terms of how best to present information. These challenges arise from the large amount of evidence emerging from the decision sciences and behavioural economics that demonstrates how difficult it is to avoid influencing decisions in one direction or another [43,45-47], given a) the large number of biases that are induced by how information is framed, and b) the fact that information must be framed one way or another. Further research is needed to identify ways of minimising biases in the presentation of the information and options, and to develop best practice recommendations for achieving balance in PtDAs.

### Future research on ways of enhancing balance

The findings of this review suggest that presentation of information in a side-by-side display format is associated with increased perceptions of “balance” among PtDA users. However, a number of questions about the ways of enhancing balance still remain unanswered and warrant further investigation. These are outlined below.

#### What are the different ways in which balance of information and options can be enhanced?

This review addressed only one of the potentially useful ways of improving the balance—that is, the use of a side-by-side presentation format. Further research is needed to identify, compare, and evaluate different ways of enhancing balance.

#### Do different types of presentation formats lead to differences in ratings of balance?

In particular, are across-group differences in balance ratings provided by people receiving PtDAs with side-by-side display of evidence information, side-by-side display of argument information, or side-by-side displays of both evidence and argument?

#### Are different techniques needed to enhance the balance of content and format?

Side-by-side presentation of information strives to achieve a balanced description of options and their attributes as it enables people to compare and contrast the options. However, it does not address the extent to which the content of the information and the probability formats used in the side-by-side presentation are themselves balanced. This leaves the possibility that information within the side-by-side presentation may still be unbalanced. This possibility creates challenges for PtDA developers in terms of identifying attributes of options, selecting evidence or arguments to include, deciding on the level of detail, deciding under which option to mention an argument, assessing the quality of underlying evidence, and choosing a format for presenting the information. Side-by-side display of evidence may be more structured than that of arguments—possibly entailing fewer opportunities for bias—but evidence may be inconsistent, lacking in precision, or simply not available, and agreement about evidence may be hard to obtain. Further research is required to provide guidance on how to develop PtDA-provided information that is balanced in content as well as in format.

#### How effective are different approaches to enhancing balance?

This review has assessed the impact of a side-by-side display format on people’s perceptions of balance; however, it is not clear whether such a display format also enables people to process the information without bias and engage in more informed decision making. Future studies investigating ways of enhancing balance of information and options should assess whether the techniques also improve the quality of information processing and decision making.

## Limitations

We acknowledge that our review has some limitations. The review was restricted to articles published in English, only two electronic databases were included in the search strategy, and dual screening of search results was only partially performed. These limitations may have inadvertently narrowed the search process and excluded potentially relevant articles. It is acknowledged that the resultant pool of reviewed articles from which the findings were derived was relatively small.

## Conclusion

Our review broadened the definition of “balance” to include complete and unbiased presentation of the relevant options and the information about those options—in content and in format—in a way that enables individuals to process this information without bias. PtDAs should present the options and information about the options in a balanced manner in order to minimise the biases in patients’ decision making that may be inadvertently introduced by the way in which information is presented—in content, as well as in format. The review of empirical evidence on assessment of balance suggests that side-by-side presentation of information and options in PtDAs is associated with increased perception of balance; however, little is known about how best to present the content and probability format of the information to achieve such balance. A number of recommendations are available from the decision sciences on how best to present information in order to improve patients’ understanding and de-bias the information context [10,57,58]. Further research is yet required to investigate different ways of enhancing the balance in the presentation of information.

## List of abbreviations used

ANOVA: univariate analysis of variance; IPDAS: International Patient Decision Aid Standards; PSA: prostate-specific antigen; PtDAs: patient decision aids; RCTs: randomised controlled trials; SPSS: Statistical Product and Service Solutions

## Competing interests

PS, PA, PB, EO, JBB, AB, DCV and RV declare that they have no competing interests.

NC has received reimbursements from educational companies that develop and test decision aids, including Expert Medical Navigation Inc (Chief Scientific Officer) and EmmiSolutions LLC (Adviser) and Janssen Scientific Affairs, LLC (adviser). She holds a patent (pending) for a computer-based decision support tool.

## Authors’ contributions

PA made substantial contributions to conception and design, acquisition of data, analysis and interpretation of data, was involved in drafting the manuscript or revising it critically for important intellectual content, and gave final approval of the version to be published.

RJV made substantial contributions to conception and design, acquisition of data, analysis and interpretation of data, was involved in drafting the manuscript or revising it critically for important intellectual content, and gave final approval of the version to be published.

JBB made substantial contributions to conception and design, acquisition of data, analysis and interpretation of data, was involved in drafting the manuscript or revising it critically for important intellectual content, and gave final approval of the version to be published.

PB made substantial contributions to conception and design, acquisition of data, analysis and interpretation of data, was involved revising the manuscript critically for important intellectual content, and gave final approval of the version to be published.

AB made substantial contributions to conception and design, acquisition of data, was involved revising the manuscript critically for important intellectual content, and gave final approval of the version to be published.

EO made substantial contributions to acquisition of data, analysis and interpretation of data, was involved revising the manuscript critically for important intellectual content, and gave final approval of the version to be published.

DCV made substantial contributions to acquisition of data, analysis and interpretation of data, was involved revising the manuscript critically for important intellectual content, and gave final approval of the version to be published.

NC made substantial contributions to conception and design, acquisition of data, analysis and interpretation of data, was involved revising the manuscript critically for important intellectual content, and gave final approval of the version to be published.

PS made substantial contributions to conception and design, acquisition of data, analysis and interpretation of data, was involved in drafting the manuscript or revising it critically for important intellectual content, and gave final approval of the version to be published.

## Supplementary Material

Additional file 1 **Table S1:** Definitions, Rationales and Methodological Comments about Balancing the Presentation of Options and Information in Patient Decision Aids Click here for file

Additional file 2 **Table S2:** Summary of Patient Decision Aid Studies in which the Balance of the Aid was assessed Click here for file

## Declarations

Publication of this supplement was funded by an unrestricted grant from the Informed Medical Decisions Foundation and by the University of Stirling (Abhyankar). Administrative and editorial support for this supplement was also provided by The University of Texas MD Anderson Cancer Center.

This article has been published as part of *BMC Medical Informatics and Decision Making* Volume 13 Supplement 2, 2013: The International Patient Decision Aid Standards (IPDAS) Collaboration’s Quality Dimensions: Theoretical Rationales, Current Evidence, and Emerging Issues. The full contents of the supplement are available online at http://www.biomedcentral.com/bmcmedinformdecismak/supplements/13/S2.
