# Supplementary material for: Balancing the presentation of information and options in patient decision aids: an updated review
Source: BMC Med Inform Decis Mak. 2013 Nov 29;13(Suppl 2):S6. doi: 10.1186/1472-6947-13-S2-S6 (PMC4044010; doi:10.1186/1472-6947-13-S2-S6)
Supplement: Additional file 1 — Table S1: Definitions, Rationales and Methodological Comments about Balancing the Presentation of Options and Information in Patient Decision Aids [file 1472-6947-13-S2-S6-S1.pdf]

Table S1. Definitions, Rationales, and Methodological Comments about Balancing the Presentation of Options and Information in Patient Decision Aids

Author, Year, Citation	Terms Used To Describe the “Balancing” Quality Dimension	Definition of “Balance”	Theoretical Rationale for Balance	Ways of Enhancing Balance
Elwyn, O’Connor et al, 2006 [1]	PtDAs are unbiased	N/A	N/A	Use of patient stories in PtDAs is best avoided until their impact is better understood, as these could introduce bias due to self-identification.
Elwyn, O’Connor et al, 2009 [2]	N/A<br>The paper describes the development of IPDASi. The paper only mentions that balance is incorporated into the information dimension of IPDASi; there is no description of what constitutes balance.		N/A	N/A
Evans, Elwyn et al, 2007 [3]	Balance of the information	Equal emphasis on positive and negative information	N/A	Presenting contrasting information, opinions and experiences.
Feldman-Stewart, Brennenstuhl et al., 2007 [4]	Accuracy<br>Balance / Imbalance	The information must be relevant, accurate, updated and complete.	N/A	1. Describing treatment procedures for each option.<br>2. Giving equal emphasis to false positives and false negatives.<br>3. Labeling numeric values as estimates and providing further information about uncertainty upon patients’ request.<br>4. Providing citations to information.

				5. Patient narratives to be avoided until the potential biasing effect is better understood.
Griffith, Fichter et al., 2008 [5]	Clarity and balance	Subjective measurement of PtDAs which avoids inclination to one decision.	N/A	For screening PtDAs, including a “no screening option”.
Martin, Brower et al., 2012 [6]	Unbiased (accurate) information	Accurate recall of information.	N/A	Including graphical images that provide a visual representation of numbers (e.g., pictogram or speedometer).
Roberts, Raynes-Greenow et al., 2004 [7]	Unbiased information<br>Non-directive	No influence on the uptake of either option.	Informed decision making requires that information is unbiased, based on current high quality evidence, gives a balanced view and does not ignore uncertainties and scientific controversies.	N/A

Ubel, Smith et al., 2010 [8]	Neutrality	-Absence of cognitive biases<br>-Neutrality means avoiding value judgments in the information (e.g., suggesting through information presentation that risks or benefits are more important)<br>-Placing equal weight on equally important information.	Cognitive biases resulting from the order in which information is presented (i.e. recency and primacy effects) negatively affect knowledge, comprehension, perception of benefits and anxiety about side-effects. Neutrality of information is essential to minimize such cognitive biases.	Presenting contextual risk information (e.g., information on competing risks the patients would face over the next five years, such as risks of experiencing colon cancer, a heart attack, or all-cause mortality).
Wills, Holmes-Rovner, 2003 [9]	Complete and balanced treatment-related information	Not explicitly defined	Creating realistic expectations about choice consequences, improving understanding of probability information, and clarity about personal	1. Presenting probabilities as ‘‘natural frequencies;’’ e.g., ‘‘1 in 10 people’’.<br>2. Use of absolute risk descriptions

			values are goals of balanced information provision.<br>When information is not complete and balanced, people may ignore missing information, devalue a treatment option partially or completely, or make inferences about unavailable information based on the information they do have.<br>Some aspects of comprehension may be influenced by information processing tendencies that are naturally associated with the central nervous system structure/function of humans.	and by provision of contextual risk information.<br>3. Tailoring the format of risk communication to an individual's level of numeracy.<br>4. Presenting information in both positive and negative frames.<br>5. Use of graphics to present numerical probability information. However, some formats may result in misunderstanding of information.<br>6. Placing information in context; ‘‘risk ladders’’ and ‘‘action standards’’ may serve as reference points.

Winterbottom, Bekker et al, 2008 [10]	Balanced information about the advantages and disadvantages of all the treatment options.	Presentation of information in a way that enables individuals to process this information without bias.	Rationale offered in terms of the heuristic/systematic information processing model. Patient narratives likely to encourage the use of heuristic processing. The context of the message, such as who is delivering the information, becomes more influential than the message content.	Use of patient narratives should be treated cautiously until their impact is better understood.
Zapka, Geller et al, 2006 [11]	Complete and clear information; bias in selection and presentation of information	Not explicitly defined	No rationale provided other than stating that “what facts are presented to women about screening and how information is presented, is basic to informed decision-making”.	N/A
Zikmund-	Balanced	Presenting specific probability	Patients have a natural inclination to	Presenting risk information in graphic

Fisher, Ubel et al., [12]	presentation of risks and benefits	information regarding both good and bad health outcomes of their decisions and by describing these outcomes in imaginable and identifiable formats.	focus on the benefits of potential medical treatments. Balanced presentation of risks and benefits is essential as it leads to better comprehension and guards against undesirable biases.	format (e.g., pictographs)

References

[1] Elwyn G, O'Connor A, Stacey D, Volk R, Edwards A, Coulter A, Thomson R, Barratt A, Barry M, Bernstein S, Butow P, Clarke A, Entwistle V, Feldman-Stewart D, Holmes-Rovner M, Llewellyn-Thomas H, Moumjid N, Mulley A, Ruland C, Sepucha K, Sykes A, Whelan T: **International Patient Decision Aids Standards (IPDAS) Collaboration. Developing a quality criteria framework for patient decision aids: online international Delphi consensus process.** *BMJ* 2006, **333**: 417-419.

[2] Elwyn G, O'Connor AM, Bennett C, Newcombe RG, Politi M, Durand MA, Drake E, Joseph-Williams N, Khangura S, Saarimaki A, Sivell S, Stiel M, Bernstein SJ, Col N, Coulter A, Eden K, Härter M, Rovner MH, Moumjid N, Stacey D, Thomson R, Whelan T, van der Weijden T, **Edwards A: Assessing the quality of decision support technologies using the International Patient Decision Aid Standards instrument (IPDASi).** *PLoS One* 2009; **4**: e4705.

[3] Evans R, Elwyn G, Edwards A, Watson E, Autosker J, Grol R: **Toward a model for field-testing patient decision-support technologies: a qualitative field-testing study**. *J Med Internet Res* 2007; **9**: e21.

[4] Feldman-Stewart D, Brennenstuhl S, McIssac K, Austoker J, Charvet A, Hewitson P, Sepucha K , Whelan T: **A systematic review of information in decision aids.** *Health Expect* 2007; **10**: 46-61.

[5] Griffith J, Fichter M, Fowler F, Lewis C, Pignone M: **Should a colon cancer screening decision aid include the option of no testing? A comparative trial of two decision aids.** *BMC Med Inform Decis Mak* 2008; **8**: 10.

[6] Martin R, Brower M, Geralds A, Gallagher P, Tellinghuisen D: **An experimental evaluation of patient decision aid design to communicate the effects of medications on the rate of progression of structural joint damage in rheumatoid arthritis.** *Patient Educ Couns* 2012; 86: 329-334

[7] Roberts CL, Raynes-Greenow CH, Nassar N, Trevena L, McCaffery K: **Protocol for a randomised controlled trial of a decision aid for the management of pain in labour and childbirth** [ISRCTN52287533]. *BMC Pregnancy Childbirth* 2004; **4**(09).

[8] Ubel P, Smith D, Zikmund-Fisher BJ, Derry H, McClure J, Stark A, Wiese C, Greene S, Jankovic A, Fagerlin A: **Testing whether decision aids introduce cognitive biases: results of a randomized trial.** *Patient Educ Couns* 2010; **80**: 158-163.

[9] Wills CE and Holmes-Rovner M: **Patient comprehension of information for shared treatment decision making: State of the art and future directions**. *Patient Educ Couns* 2003; **50**: 285-290.

[10] Winterbottom A, Bekker H, Conner M, Mooney A: **Does narrative information bias individual's decision making? A systematic review**. *Soc Sci Med* 2008; **67**: 2079-2088.

[11] Zapka JG, Geller BM, Bulliard JL, Fracheboud J, Sancho-Garnier H, Ballard-Barbash R; IBSN Communications Working Group: **Print information to inform decisions about mammography screening participation in 16 countries with population-based programs.** *Patient Educ Couns* 2006; **63**: 126-137.

[12] Zikmund-Fisher BJ, Ubel PA, Smith DM, Derry HA, McClure JB, Stark A, Pitsch RK, Fagerlin A: **Communicating side effect risks in a tamoxifen prophylaxis decision aid: the debiasing influence of pictographs**. *Patient Educ Couns* 2008; **73**: 209-214.
