# Supplementary material for: Balancing the presentation of information and options in patient decision aids: an updated review
Source: BMC Med Inform Decis Mak. 2013 Nov 29;13(Suppl 2):S6. doi: 10.1186/1472-6947-13-S2-S6 (PMC4044010; doi:10.1186/1472-6947-13-S2-S6)
Supplement: Additional file 2 — Table S2: Summary of Patient Decision Aid Studies in which the Balance of the Aid was assessed [file 1472-6947-13-S2-S6-S2.pdf]

Table S2. Summary of Patient Decision Aid Studies in Which the Balance of the Aid was Assessed

**Author, Year, Citation**	**Decision context and how balance was considered.**	**Definition of "balance".**	**Whose perspective was assessed?**	**Response categories for assessing balance.**	**Description of side-by-side display format for contrasting options.**	**Results: How many rate PtDA as "balanced"?**
Mathieu, Barratt et al., 2010 [1]	Effect of PtDA on knowledge and ability to make informed decision about mammogram screening at age 40.<br><br>"(N)eed for balanced, evidence-based information that enables informed decision making."	No explicit definition, but explains how to achieve balance.<br><br>Present cumulative risks over an appropriate time frame, use diagrams for probabilities, use large denominators, show event rates as whole numbers, and provide context to consider the effect.	Women aged 38-45 who accessed the website and met eligibility criteria (117 completed the acceptability questions).	5-point Likert scale:<br>• Clearly towards not screening<br>• A little towards not screening<br>• Completely balanced<br>• A little towards having screening<br>• Clearly towards having screening	No side-by-side display format included.	49% (57) rated PtDA as completely balanced.<br><br>29% (34) rated PtDA as a little towards not screening.

Smith, Trevena et al, 2010 [2]	Balanced and fair information on bowel cancer screening.	No definition offered.	334 adults aged between 55 and 64 with low education attainment eligible for bowel cancer screening.	Not mentioned.	No side-by-side display format; risks on separate pages, with mix of two- and multi-colour risk figures.	48% (160) rated PtDA as completely balanced.
Griffith, Fichter et al., 2008 [3]	Primary measures: clarity and balance comparing video PtDA versions with and w/o explicit discussion of option to not be screened.	No definition offered.	Single sex volunteer focus groups. 12 groups at 3 sites, total of 106.	5-point scale:<br>• Strongly in favor of screening<br>• Somewhat in favor<br>• Neither in favour nor against screening<br>• Somewhat against<br>• Strongly against	No side-by-side display formats were used (according to author).<br><br>Mentions comparison of the different tests; not clear if the “no screen” option is in the comparison.	16% rated PtDA is neither in favour nor against screening, for both versions.

Spunt, Deyo et al., 1996 [4]	Balance: completely balanced (if the patients found the program to be balanced).	No definition offered.	239 patients affected with low back pain.	5-point scale:<br>• Clearly slanted—surgery<br>• Slightly slanted—surgery<br>• Completely balanced<br>• Slightly slanted--nonsurgical therapy<br>• Clearly slanted--nonsurgical therapy	Author did not respond to query about use of side-by-side display format.	56% (133) rated PtDA “completely balanced.”
Anderson, Carter et al., 2011 [5]	Equally emphasized downsides and benefits.	No definition offered.	22 women with ovarian cancer.	5-point scale: Decision aid equally emphasized downsides and benefits:<br>• Strongly disagree<br>• Disagree<br>• Neutral<br>• Agree<br>• Strongly agree	Side-by-side display format includes benefits, side effects arguments, with some numbers.	17 of 19 participants (89%) strongly agreed or agreed.

Watson, Hewitson et al., 2006 [6]	RCT of a PtDA about PSA testing. Assessed whether the information in the aid was presented in a “balanced way.”<br><br>Also used a measure of decisional balance as a measure of attitudes toward PSA testing.	No definition offered.	Men from 11 GP practices, recruited through computerized registries.	Not fully stated. One option was, information was presented in a “balanced way.”	Aid included a summary of the potential benefits and downsides of testing. Information presented as a side-by-side figure/ table.	94% of 468 respondents rated the information in the PtDA as presented in a “balanced way.”
Drake, Engler-Todd et al., 1999 [7]	Open-ended question concerning fairness and balance during pilot test; in evaluation trial close-ended questions also concerning balance and fairness.	No definition offered. Implied that balance was equivalent to fairness.	21 women of advanced maternal age and 17 spouses.	5-point scale:<br>• Clearly slanted to testing<br>• Slightly slanted to testing<br>• Completely balanced<br>• Slightly slanted to no testing<br>• Clearly slanted to no testing	Worksheet format with risks presented using icon arrays.	84% women (n = 21) and 53% men (n = 17)<br><br>chose “completely balanced.”

Lalonde, O’Connor et al., 2004 [8]	Development of a PtDA to improve decision quality and adherence to chosen option.	No definition offered. Provided “balanced examples of how others go through the steps” (of making the decision)	16 patients receiving pharmacologic treatment for hypertension and/or dyslipidemia.	3-point scale:<br>• Slanted towards lifestyle options<br>• Slanted toward taking drugs<br>• Balanced	Risk of heart disease and stroke presented next to benefits using worksheet format.	80% rated PtDA as balanced (12 of 16).
van Tol-Geerdink, Stalmeier et al., 2006 [9]	Was the information presented in a structured and balanced way?	No definition offered.	150 prostate cancer patients.	5 categories:<br>• Clearly in favor of lower dose<br>• Somewhat in favor of lower dose<br>• Balanced<br>• Somewhat in favor of higher dose<br>• Clearly in favor of higher dose	Probabilities of outcomes presented side-by-side using pie charts.	95% (142) reported “balanced” in PtDA group.

van Tol-Geerdink, Leer et al. submitted [10]	Was the information presented in a structured and balanced way?	No definition offered.	240 patients with prostate cancer choosing between surgery or radiotherapy.	4 categories:<br>• Yes, the information was balanced<br>• No, in favour of surgery<br>• No, in favour of brachytherapy<br>• No, in favour of external beam radiotherapy	Probabilities of outcomes presented in an evidence table using pie charts.	96% (147 of 153) reported “balanced” category.

References

[1] Mathieu E, Barratt AL, McGeechan K, Davey H, Howard K, Houssami N: **Helping women make choices about mammography screening: an online randomized trial of a decision aid for 40-year-old women.** *Patient Educ Couns* 2010; **81**: 63-72.

[2] Smith S, Trevena L, Simpson J, Barratt A, Nutbeam D, McCaffery K: **A decision aid to support informed choices about bowel cancer screening among adults with low education: randomised controlled trial.** *BMJ* 2010; **341**: c5370

[3] Griffith J, Fichter M, Fowler F, Lewis C, Pignone M: **Should a colon cancer screening decision aid include the option of no testing? A comparative trial of two decision aids.** *BMC Med Inform Decis Mak* 2008; **8**: 10.

[4] Spunt B, Deyo RA, Taylor VM, Leek KM, Goldberg H, Mulley A: **An interactive videodisc program for low back pain patients.** *Health Educ Res* 1996; **11**: 535-541.

[5] Anderson C, Carter J, Nattress K, Beale P, Philp S, Harrison J, Juraskova I: **The booklet helped me not to panic": A pilot of a decision aid for asymptomatic women with ovarian cancer and with rising CA-125 levels.** *Int J Gynecol Cancer* 2011; **21**: 737-743.

[6] Watson E, Hewitson P, Brett J, Bukach C, Evans R, Edwards A, Elwyn G, Cargill A, Autoske J: **Informed decision making and prostate specific antigen (PSA) testing for prostate cancer: A randomised controlled trial exploring the impact of a brief patient decision aid on men's knowledge, attitudes and intention to be tested.** *Patient Educ Couns* 2006; **63**(SPEC. ISS 3): 367-379.

[7] Drake E, Engler-Todd L, O'Connor AM, Surh LC, Hunter A: **Development and evaluation of a decision aid about prenatal testing for women of advanced maternal age.** *J Genet Couns* 1999; **8**: 217-233.

[8] Lalonde L, O'Connor AM, Drake E, Duguay P, Lowensteyn I, Grover SA: **Development and preliminary testing of a patient decision aid to assist pharmaceutical care in the prevention of cardiovascular disease.** *Pharmacotherapy* 2004; **24**: 909-922.

[9] van Tol-Geerdink JJ, Stalmeier PF, van Lin EN, Schimmel EC, Huizenga H, van Daal WA, Leer JW: **Do prostate cancer patients want to choose their own radiation treatment?** *Int J Radiat Oncol Biol Phys* 2006; **66**: 1105-1111.

[10] van Tol-Geerdink JJ, Leer JW, Wijburg C, van Oort IM, Vergunst H, van Lin EJNT, Witjes JA, Stalmeier PFM. **Patient participation in the treatment choice does not induce regret: RCT of a decision aid on the treatment choice for prostate cancer.** (submitted).
